# Single-Molecule Study of Proteins by Biological Nanopore Sensors

**DOI:** 10.3390/s141018211

**Published:** 2014-09-29

**Authors:** Dongmei Wu, Sheng Bi, Liyu Zhang, Jun Yang

**Affiliations:** 1 College of Pharmacy, Jiamusi University, Jiamusi 154007, China; E-Mail: zhangliyuzly@gmail.com; 2 The Affiliated First Hospital of Jiamusi University, Jiamusi 154007, China; E-Mail: Yangj2004@126.com

**Keywords:** α-hemolysin, protein conformation, protein-ligand interactions, new nanopores

## Abstract

Nanopore technology has been developed for detecting properties of proteins through monitoring of ionic current modulations as protein passes via a nanosize pore. As a real-time, sensitive, selective and stable technology, biological nanopores are of widespread concern. Here, we introduce the background of nanopore researches in the area of α-hemolysin (α-HL) nanopores in protein conformation detections and protein–ligand interactions. Moreover, several original biological nanopores are also introduced with various features and functions.

## 1. Introduction

Nanopore sensors are a detection technology for a rapid and label-free single molecule analysis in the field of analytical chemistry. In the case of provided voltage, an analyte is driven towards the nanopore producing a measurable ionic–current blockade. Analyzing the characteristic currents, durations, frequencies and even the shapes of blockades reveals the properties of analytes [1]. Nanopore technology has been widely applied to analysis of single molecules including RNA and DNA sequencing with the advantages of detection in high-throughput, and in most cases, no requirement for labeling/immobilization in the biomedical field [2]. Nanopore technology can be roughly divided into two fields: biological nanopore technology and solid-state nanopore technology. Due to the fact that diameter, length and surface of solid-state nanopores are controllable, solid-state nanopores are often used for detection of larger biomolecules [3–5]. However, the intensive electrical noise is the most serious disadvantage for solid-state nanopores [6–8]. Contrary to solid-state nanopores, biological nanopores have a well-controlled geometry and excellent reproducibility and high sensitivity, a more preferable signal-to-noise ratio for single molecule analysis. Based on the advantages, biological nanopore technology has been paid more attention by scientists.

Proteins participate in a wide variety of physiological activities inside living cells. Elucidating the properties of proteins and evaluating their functional roles will benefit biomedical research. Many reported methods of the laboratory, including immunoassay, HPLC-MS and western blotting *etc.*, can perform accurate detections for proteins, however, they are generally too slow to conduct a rapid response [2]. Biological nanopore measurement is real-time, sensitive, selective and stable; these outstanding features have led to numerous researches of proteins in the single-molecule level by nanopore techniques. Hence, this mini review focuses on detecting proteins in the single-molecule level by biological nanopores.

## 2. α-Hemolysin Nanopores

**Figure 1.**
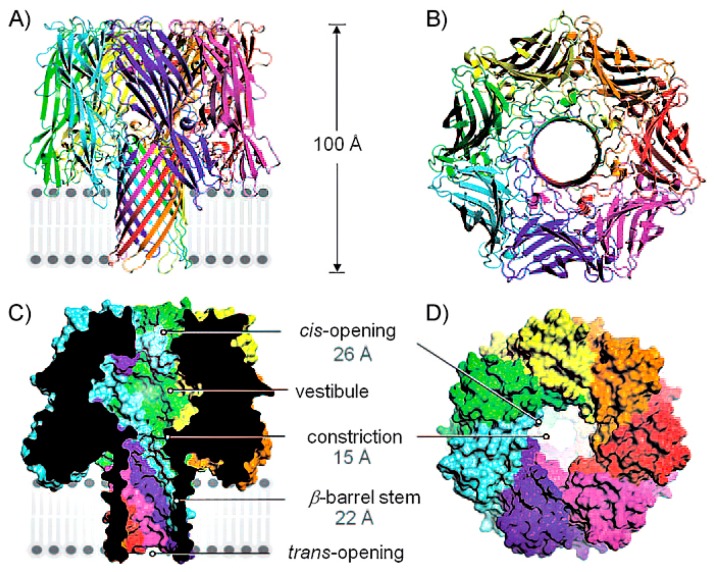
(**A**) Observe the position between the α-HL nanopores with the lipid bilayer; (**B**) View of α-HL from the cis side to the pore; (**C,D**) The cross section and the space filling model show that the hole diameter of α-HL in different locations. Reproduced with permission from [10].

In 1996, it was reported that ssDNA and RNA could be actuated electrophoretically through an α-hemolysin (α-HL) nanopore [9]. The α-hemolysin (α-HL) nanopore has been the most commonly used recently because of its appropriate inner diameter and repeatable self-assembly ability (Figure 1). The hydrophilic α-HL nanopore which arises from Staphylococcus aureus can self-assemble into planar lipid membrane. α-HL nanopore is of an asymmetric, mushroom shape. The pore is mainly composed of three structural domains. Firstly, the stem is the β-barrel structure that perforates the membrane; secondly, the rim is the lipid membrane anchoring domain; thirdly, the cap is the external part. The narrowest diameter, the widest diameter and the height of pore are 1.4 nm, 4.6 nm and 10 nm, respectively [6].

An α-HL is located in a planar lipid membrane that separates two compartments which are called *cis* and *trans.* Electrolyte buffer is added into each compartment. Two integrated electrodes are installed to form the constant potential which is used for driving the analytes to thread through the pore. The experiments are carried out with high concentration salt solution (1.0 M KCl) instead of a physiological one in the α-HL-based protein detections. The mean conductance for the α-HL pore shows a linear relationship with the conductivity of the electrolyte solution at a given potential [4]. An analyte is driven towards the biological membrane pore by an applied potential establishing a measurable ionic–current blockade. The ionic current is interrupted when an analyte enters into the α-HL pore at a given potential. Analyzing the characteristic blockade currents, durations, frequencies and the shapes of blockades reveals the properties of the analyte, such as, size, conformation, structure, charge, geometry and interactions.

## 3. Protein Conformation Detection with Biological Nanopores

Since the first paper for biological nanopore-based peptide analysis was published in 2004 [11], scientists have seen great achievements in the field of single-molecule detection of proteins. Most of the biological nanopore study on protein detections used α-HL to construct a translocation system. The α-HL could capture the protein or peptides in their varied conformations under the applied potential. For example, Jeremy S. Lee and co-workers [11] synthesized the peptides containing different repeats of the collagen-like sequence (Gly-Pro-Pro)_n_ (*n* = 1 P_1_, *n* = 2 P_2_, and *n* = 3 P_3_) that is terminated in ferrocene through nanopores. Peptides which contain more repeats can give larger blockade current and duration. Analysis of the characteristic contour plots can distinguish their conformation as single, double, or triple helices.

ssDNA molecules are short enough to pass through the pore, and results in brief, measurable blockades of the current. Binding of protein to ssDNA allows capturing but not translocating because the protein bound at one end is larger than the pore diameter. Driving of single DNA molecules through α-HL enables the rapid measurement of DNA–protein interactions at a given potential [12].

Peptide–oligonucleotide conjugates have attracted great attention in the biomedical field. Yi-Tao Long and co-workers [13] studied the process which peptide–oligonucleotide conjugates translocating α-HL nanopores. The result showed that increasing length of the two peptide–oligonucleotide conjugates resulted in the increase of durations. In addition, the structure of peptide–oligonucleotide conjugates could be discriminated by α-HL nanopore at the single-molecule level [13].

**Figure 2.**
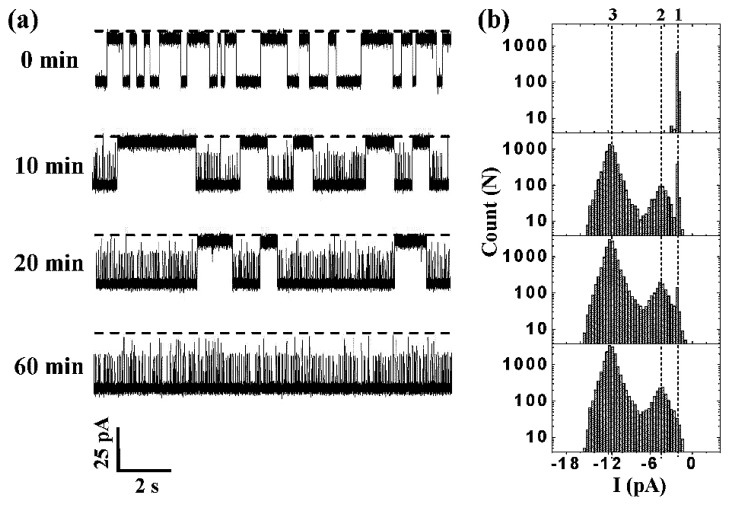
Detecting of A-β(10-20) cleavage by trypsin. (**a**) Single channel at different times of the recording track representative fragments. Dashed lines mean zero current; (**b**) Histograms of corresponding time-dependent event amplitude. The mean residual current levels of peptides YEVHHQKLVFF, YEVHHQK, and LVFF are represent by dashed lines 1, 2, and 3. Reproduced with permission from [15].

Through research of the protein, scientists found it is difficult to detect some large peptide or protein molecules through the α-HL. A peptide or protein molecule was attempted to be cleaved by the enzyme to detect the structure and the enzymatic cleavage of polypeptides. Trypsin is widely used in the cleavage of the polypeptides *in vivo* and *in vitro.* Trypsin can specifically identify the polypeptides containing Arg or Lys residues under certain conditions [14]. Guan and co-workers [15] take advantage of the characteristics of cleavage of the trypsin to analyze the composition of polypeptide and cleavage of polypeptides through the α-HL (Figure 2) [15]. Trypsin is too large to pass through α-HL and cannot cause blocking current. However, the polypeptides which are sufficiently small to pass through the hole and generate a corresponding blocking current are cleaved. The data of current blockades under different cleavage times can reveal the composition of polypeptides and the process of peptide cleavage. This approach may be a basis for research on polypeptides in the future.

Many factors affect the nanopores during the translocation process, such as the applied potential, temperature and the composition of solution. Wang and co-workers [16] found that the bumping events could be generated via regulating the applied potential across the membrane. α-syn is critical in the pathogenesis of Parkinson's disease. The natively unfolded α-syn monomer can traverse through the α-HL pore under the potential of +100 mV. When the potential is higher than +100 mV, a partially folded intermediate would be captured inside the vestibule of the α-HL. At +70 mV, blocking current decreases further in order to produce the intermediate. After lowering the voltage to +40 mV, the captured intermediate exited from the vestibule of the α-HL nanopore. This phenomenon reveals that the intermediate which undergoes structural transformation of α-syn is involved in the potential [16].

Temperature is another factor which has an effect on the detection process of nanopores. Amit Meller and co-workers [1] demonstrated the strong correlation between temperature and the conformation changes of some polymers. The poly (dA) remains as two separate state from 15.0 to 40.0 °C. However, for the poly (dC), when the temperature is 20 °C, it falls into two groups. When the temperature is above 25 °C, it shows a widely separated state. This experiment confirmed that the temperature is an important factor which has different effects on different polymers; temperature affected inhibition and aggregation of polymers [1].

The composition of resolution can affect the translocation process of nanopores. α-synuclein is an important protein, closely related to the pathogenesis of Parkinson's disease and other neurodegenerative diseases. Unfolded α-synuclein monomer can be converted into partially folded intermediate after fibrillation under intrapeptide electrostatic interaction. Trehalose can change the surface hydrophobic interaction of A53T α-synuclein. Unfolded α-synuclein monomer can translocate through an α-HL nanopore and generate blocking current. Partially folded intermediate captured by the α-HL can produce capture current. Yi-Tao Long and co-workers [16] demonstrated trehalose was involved in protein secondary structure by inhibiting the interpeptide interaction. Trehalose is expected to become an effective drug treatment of neurodegenerative diseases [16].

**Figure 3.**
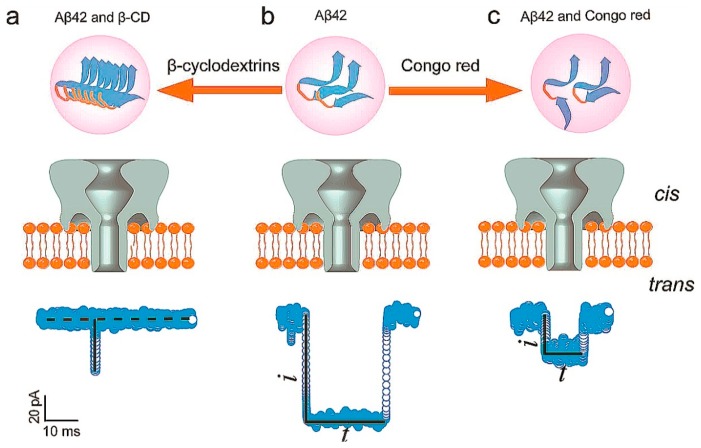
Aβ42 was added from the *cis* chamberin with and without β-cyclodextrin (Aβ42-CD) or Congo Red (Aβ42-CR), respectively. (**a**) Manifestation of the collision event of Aβ42-CD; (**b**) Manifestation of the blockage event of Aβ42; (**c**) Manifestation of the blockage event of Aβ42-CR. Reproduced with permission from [17].

β-Amyloid 42 (Aβ42) is an important peptide which can deposit in the nerve cells. It is of great significance in the diagnosis and treatment of Alzheimer's disease (AD). At present, there are many detection methods to Aβ42; nanopore technology can also be applied to the detection of Aβ42. Aβ42 has three phases as monomers, protofibrils, and fibrils in nerve cells. Aβ42 can generate blocking current caused by a translocation event. β-cyclodextrins (β-CD) which act as promoters can promote monomers to form short and thick Aβ42 fibrils. Congo red (CR) which act as inhibitors could inhibit the aggregation of Aβ42. Monomers and protofilaments can cause different duration times when they translocate through the nanopore. The reduced aggregated fibrils formation was caused by the binding of CR and Aβ42. When Aβ42 and CR were added into cis chamber, blocking current and duration revealed fewer aggregated fibril formations. When the β-CD were added into chamber, blocking current and duration revealed opposite results. The interaction between Aβ42 and CR or β-CD can provide a basis for future research to Aβ42 (Figure 3) [17].

## 4. Probing Protein–Ligand Interactions

In addition to straight detecting protein, α-HL nanopore is also widely used to probe protein–ligand interactions. One of the strategies is to attach aptamer at the entrance or the lumen of α-HL. By using this engineered α-HL, the region for the recognition of individual protein molecules is shift from the cavity of the α-HL to the outside of the channel. This strategy provides a novel method to allow the single-molecule study of large protein by α-HL. Moreover, searching for the alternative membrane protein is another promising solution to broaden the applications of nanopore techniques.

The cysteine residues in α-HL nanopore can link to an DNA or RNA oligonucleotide which is capable of bind various analytes, including small molecules, proteins, and even cells. Dvir Rotem and co-worker [18] used modified α-HL nanopore with a 15-mer DNA aptamer to detect thrombin. The cation-stabilized quadruplex composed of thrombin and aptamer was detected and analyzed. The data of blocking current and duration can reveal concentrations of thrombin, analyzing the interactions between thrombin and aptamer. The research suggested that the development of aptamers with different characteristics helped to improve the detection of protein through nanopores [18].

**Figure 4.**
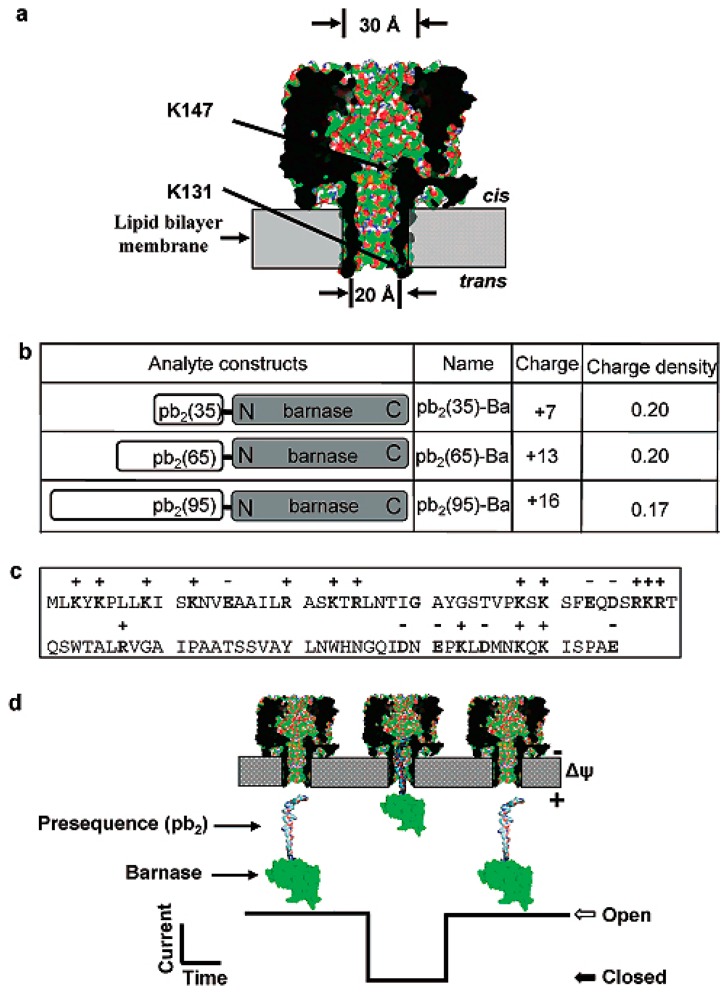
Engineered nanopores and pb_2_-Ba proteins for protein detecting: (**a**) Two mutations: K131 and K147, as shown by arrows; (**b**) Three different lengths and different charge densities of yeast precytochrome b_2_ (pb_2_) were fused to the N-terminus of barnase (Ba); (**c**) The panel represents the amino acid sequence of 95 residues; (**d**) Illustration shows how pb_2_-Ba protein divides into the nanopore from the trans side. Reproduced with permission from [19].

As we all know, the interior lumen of α-HL nanopores is hydrophobic, and the translocation process of the analyzed is related to hydrophilicity. Based on this principle, Liviu Movileanu and co-workers [19] designed α-HL nanopores in which seven amino acids was mutated to negatively charged aspartic acid residues at the trans opening. A special protein was designed as pb_2_-Ba. The protein analytes contain positively charged presequences (pb_2_) of varying length fused to the small ribonuclease barnase (Ba). Understanding the structure of proteins facilitates the research on protein–protein interactions. They demonstrated that many factors affected the interaction of the pb_2_-Ba protein with the α-HL nanopore, such as the length of pb_2_-Ba protein, the location of the electrostatic traps in α-HL nanopore, the analysis of hydrophilicity and the given potential. The presence of the electrostatic traps greatly enhanced the interaction of the pb_2_-Ba and protein of the α-HL nanopore (Figure 4) [19].

Trinitrotoluene (TNT) which is widely used in military and medical fields is an important nitroaromatic molecule. At present, nanopore technology can be applied into the detection of nitroaromatic molecules. Hagan Bayley and co-workers [20] engineered α-HL nanopores. The α-HL can be mutated at position 113, then seven aromatic side chains can project into the lumen at position 113. The engineered α-HL nanopores containing aromatic side chains can interact with nitroaromatic molecules. At a given potential, blocking current and duration can distinguish the TNT and other various nitroaromatics, and the direct aromatic–aromatic interactions can be found. The engineered nanopores could become an important way to detect nitroaromatic molecules and clarify the nature of aromatic–aromatic interactions [20].

α-HL nanopore could be used to predict the conformational changes that result from weak interactions. Ying Yilun and co-workers [21] detected the weak interaction between P53 and DNA using α-HL nanopore. Diagnostic ionic current blockages were caused by the weak interactions in the complex of p53-P and B40 (p53-P:B40) through an α-HL nanopore. It is indicated that the conformation of B40 might be changed by binding to p53-P, and the analyte–pore interactions could be enhanced by the weak interactions between p53-P and B40. So, α-HL nanopores support the possibility of identifying the weak interaction between two biomolecules at the single-molecule level [21].

## 5. New Nanopore Materials

Much progress has been made in the detection of proteins by α-HL nanopores, but there remains many challenges. The narrow diameter of α-HL is 1.4 nm, and it is difficult for some large proteins to enter into the narrow opening of α-HL. The anion selectively pass through α-HL, and the positive-charge protein needs to overcome a well-defined free energy barrier for the translocation through α-HL. To overcome the inherent geometric limitations and charge distribution of the α-HL pore, birth of new nanopore materials may encourage people to solve these problems.

A number of biological membrane proteins, including areolysin [22,23], FhuA [21], ClyA [13], hetero-oligomeric channels formed by NfpA and Nfp [9] have attracted wide attention.

The aerolysin with diameter in the range of 1 to 1.7 nm is a β-pore–forming protein secreted by *Aeromonas hydrophila.* Contrary to α-HL, the aerolysin has essentially negative global net charge. It is more suitable for studying the conformational changes of small positive-charged peptide and proteins.

Since 2006, the aerolysin nanopore was first introduced in nanopore based protein detections [24]. The aerolysin pore has been widely used for peptide translocation or protein conformational change research.

Manuela Pastoriza-Gallego and co-workers [25] used the aerolysin nanopore method to detect the wild type maltose-binding protein (MalEwt) and a destabilized variant (MalE219). They found that the transport time of unfolded proteins decreased exponentially when the applied voltage increased. Double-sized proteins which have larger volumes can cause the longer and stronger blocking current. The aerolysin nanopore have the potential to act as a tool for protein folding research in the future [25].

FhuA is a 714-residue membrane protein which is composed of 22 antiparallel-β-strands and an N-terminal 160-residue cork domain. There are 11 loops on the extracellular side and 10 short turns on the periplasmic side to link the 22 antiparallel-β-strands. FhuA contains four loops which can fold back into the lumen of nanopore. FhuA is able to maintain its stability for long periods under different conditions and show the function as transporter and receptor. Engineered FhuAΔC/Δ4L proteins include FhuA which deletes four loops and the cork domain. Liviu Movileanu and co-workers [26] verify its stability at physiological salt concentrations and in highly acidic pH conditions. Based on these characteristics, FhuA could be applied to certain conditions that mimic physiological conditions. FhuAΔC/Δ4L also can be used to detect the interaction between proteins and nanopores or other aptamers [26,27].

The ClyA nanopore, which has a crystal structure, is from Salmonella typhi. The diameter of ClyA nanopores is larger than α-HL pores. Therefore, they could distinguish larger-sized proteins. ClyA nanopores can detect the complex of analyte-specific ligands by analyzing blocking current and duration. The entrance of the ClyA nanopore can be decorated with rings of 12 protein-specific aptamers. When the protein bind to the aptamers, blockade frequencies were enhanced. Misha Soskine and co-workers [3] demonstrated that human and bovine thrombins can elicit characteristic ionic current blockades through the ClyA nanopore. The analyte which binds to the aptamers can produce its characteristic signal when it is released into the pore lumen [3].

**Figure 5.**
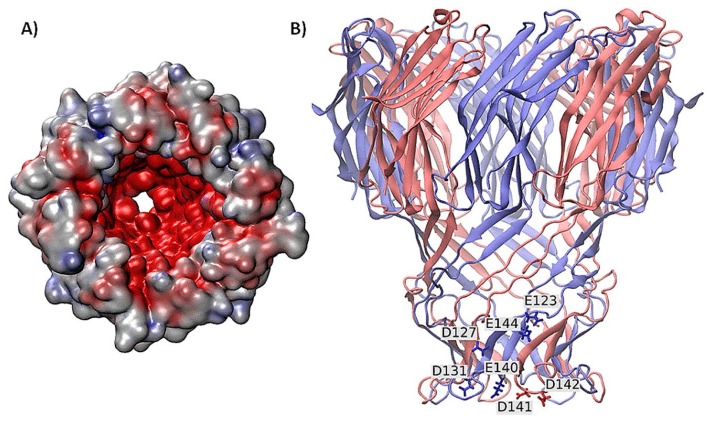
The modeled structure showed the N. farcinica porin A (NfpA) and N. farcinica porin B (NfpB) subunits. (**A**) The modeled structure with electrostatic potential surface showing highly negative potential inside the lumen of the channel; (**B**) The secondary structures of four NfpA and four NfpB subunits (red- and blue-colored). Reproduced with permission from [28].

The channel which is derived from the Gram-positive bacterium Nocardia farcinica is the hetero-oligomer whose structure is related to MspA of M. smegmatis. Two subunits, NfpA and NfpB, were reconstituted in lipid bilayers. The channel shows highly negative potential caused by negatively charged amino acid residues clustering in the channel. The channel causes asymmetric transport properties which is caused by asymmetric structure under applied potential. When peptide enter into the channel and data of blocking current and duration can reveal detailed kinetic information. The kinetic data obtained can be used to distinguish the peptide binding events from the translocation events. The channel has good prospects to detect protein in the future (Figure 5) [28]. The different types of biological nanopores are summarized and listed in Table 1.

## 6. Conclusions

Biological nanopores are powerful tools to detect proteins at the single-molecule level. Protein molecules and protein-ligand complexes are detected by a α-HL nanopore. Methods of analyzing the electric current data are established in order to research protein conformation and interaction of proteins and ligands, protein–pore interaction and interaction of proteins and other molecules. Although much progress has been made in the detection of proteins by biological nanopores, there are still many challenges. For example, large proteins cannot enter the narrow opening of α-HL, and non-specific bumping events cannot be analyzed with nanopores. New nanopores need to be developed for detecting proteins. There are still many challenges which need to be resolved in order to use nanopores in biotechnology, medical applications and other domains.

## Figures and Tables

**Table 1.** Detection of proteins with biological nanopores.

**Analyte**	**Assisted Molecular**	**Biological Nanopore**	**Comment**	**Ref.**
Collagen-like peptide		α-HL	It can distinguish the tertiary of proteins.	[4]
Wild-type α-syn(WT α-syn) and mutant α-syn (A53T α-syn)	trehalose	α-HL	Trehalose can open A53T α-syn folded structure, but it does not work for WT α-syn.	[16]
β-Amyloid 42 (Aβ42)	β-cyclodextrin (β-CD) Congo red	α-HL	β-CD promotes the aggregation of Aβ42, but congo red inhibits the aggregation of Aβ42.	[17]
Barnase	Presequence (pb_2_) (binding)	α-HL	Barnase to pb_2_ is bound to α-HL for observing the structure of barnase.	[19]
thrombin	DNA hybridization (binding)	α-HL	This method can detect lower concentrations of thrombin, and can detect the separation of thrombin and ligand reaction.	[18]
cationic peptides		*N. farcinica porin* A (NfpA) and *N. Farcinicporin* B (NfpB)	This new type of nanopores can distinguish between characteristic signal from the peptide binding or peptide translocation.	[28]
lysozyme, (GFP-like protein)FP, (human thrombin)HT, and (bovine thrombin) BT		*Escherichia coli cytolysin* A (ClyA)	ClyA nanopore can be used to detected large folded protein.	[3]
n-dodecyl-β-D-maltopyranoside (DDM), n-octyl-β-D-glucopyranoside (OG), and 1-lauroyl-2-hydroxy-sn-glycero- 3-phosphocholine(LPhC) detergent		*Bacterial ferrichydroxamate* uptake component A (FhuA)	This nanopore maintained its stability under many experimental circumstances.	[26]
